# Breakdown of dimorphic enantiostyly is linked to reduced herkogamy and increased self-pollination

**DOI:** 10.1093/aob/mcag213

**Published:** 2026-07-17

**Authors:** Sam McCarren, Spencer C H Barrett, Alice L M Fairnie, Sinéad Watchorn, Bruce Anderson, Nicola Illing, Robert A Ingle

**Affiliations:** Department of Molecular and Cell Biology, University of Cape Town, Cape Town 7701, South Africa; Department of Biological Sciences, University of Cape Town, Cape Town 7701, South Africa; Department of Ecology and Evolutionary Biology, University of Toronto, 25 Willcocks Street, Toronto, Ontario M5S 3B2, Canada; Department of Ecology and Evolutionary Biology, University of Toronto, 25 Willcocks Street, Toronto, Ontario M5S 3B2, Canada; Paleo-Research Institute, University of Johannesburg, Johannesburg 2092, South Africa; Department of Botany and Zoology, Stellenbosch University, Stellenbosch 7602, South Africa; Department of Molecular and Cell Biology, University of Cape Town, Cape Town 7701, South Africa; Department of Molecular and Cell Biology, University of Cape Town, Cape Town 7701, South Africa

**Keywords:** Disassortative pollination, mirror-image flowers, quantum dot, reproductive assurance, selfing, homostyly, stylar polymorphism, *Wachendorfia*

## Abstract

**Background and Aims:**

The evolutionary breakdown of outcrossing mechanisms has occurred repeatedly across angiosperm lineages. Stylar polymorphisms are of particular interest because their floral morphs exhibit discrete variation with pronounced spatial separation between reproductive organs (herkogamy), a trait strongly influencing outcrossing. These polymorphisms are typically maintained by negative frequency-dependent selection resulting from disassortative (between-morph) mating. In contrast, biased morph ratios and homostylous variants with anthers and stigmas adjacent to one another often signal transitions towards self-fertilization and erosion of the polymorphism. Here, we investigate this transition in three *Wachendorfia* species that exhibit dimorphic enantiostyly. Based on observations of biased morph ratios, we predicted the breakdown of enantiostyly to be associated with reduced herkogamy and an increased likelihood of self-pollination.

**Methods:**

To test this hypothesis, we quantified morph ratios and reproductive-organ separation across 42 populations spanning the three species. In a subset of populations, we also measured flower width, pollinator visitation, seed set and pollen transfer to examine their association with floral polymorphism breakdown.

**Key Results:**

Populations with equal morph ratios typically exhibited well-developed herkogamy, consistent with disassortative mating. In contrast, reduced herkogamy, increasing the potential for autonomous self-pollination, was characteristic of populations with biased ratios or those that were monomorphic. Homostylous variants occurred in all three species and were characteristic of populations with low herkogamy and biased morph ratios as expected during early stages in the evolutionary modification of stylar polymorphism.

**Conclusions:**

Our investigation revealed that the breakdown of outcrossing polymorphisms is not restricted to heterostylous species but also occurs in species with mirror-image flowers. In both cases of breakdown, the proximate mechanism causing increased self-pollination involves changes in herkogamy, emphasizing the importance of this key reproductive trait in influencing mating-system evolution.

## INTRODUCTION

Transitions between outcrossing and self-fertilization represent one of the most frequent and consequential shifts in the reproductive modes of flowering plants (Stebbins, 1957; Barrett, 2008). These transitions shape patterns of genetic diversity, population structure and lineage diversification and are often associated with predictable changes in floral morphology, reproductive allocation, ecological interactions and demography (Lloyd, 1992; Barrett, 2002; Goodwillie *et al.*, 2005; Sicard and Lenhard, 2011; Igic and Busch, 2013; Wright *et al.*, 2013). Shifts towards self-fertilization (hereafter selfing) are commonly favoured in conditions of unreliable pollination, low mate availability and/or colonization of new habitats, where reproductive assurance can provide a short-term fitness advantage (Baker, 1955; Busch and Delph, 2012; Pannell *et al.*, 2015). Conversely, mechanisms promoting outcrossing are expected to evolve and be maintained where pollen transfer between plants is reliable and inbreeding depression high (Lande and Schemske, 1985; Charlesworth and Charlesworth, 1987). Understanding how and why these transitions occur, and identifying the traits that mediate them, remains a central goal of plant evolutionary biology.

A key floral trait influencing mating patterns is herkogamy, the spatial separation of anthers and stigmas within flowers (Breese, 1959; Opedal, 2018). By reducing interference between anthers and stigmas, herkogamy limits within-flower self-pollination, thus providing greater opportunities for outcrossing (Webb and Lloyd, 1986) Variation in herkogamy has been shown to influence selfing rates, pollen export efficiency, siring success and mate diversity within populations (Barrett and Shore, 1987; Belaoussoff and Shore, 1995; Kulbaba and Worley, 2012; Medrano *et al.*, 2012; Opedal *et al.*, 2016; Opedal, 2018). Herkogamy is a particularly labile trait that often mediates evolutionary transitions between mating systems, including the breakdown of floral polymorphisms (Barrett and Husband, 1990; Sánchez *et al.*, 2010; Brys *et al.*, 2013; Opedal *et al.*, 2017). Reductions in herkogamy are commonly associated with increased selfing and reproductive assurance (Opedal, 2018), especially when pollinators are scarce (Brys *et al.*, 2013), making it an important trait for understanding the evolutionary dynamics of mating systems.

Herkogamy reduces within-flower self-pollination but has much less influence on between-flower self-pollination (geitonogamy). Moreover, as the degree of herkogamy increases it potentially makes pollen transfer less efficient because pollen is placed and received on different parts of a pollinator’s body. Stylar polymorphisms with reciprocal reproductive organ positions have evolved multiple times in angiosperms and elegantly solve the problems of geitonogamy and reduced pollen transfer in herkogamous systems (Barrett *et al.*, 2000). Here, discrete floral morphs differing in style and anther position co-occur within populations promoting disassortative (between-morph) mating and maintaining morphs at roughly equal frequencies through negative frequency-dependent selection (Barrett and Shore, 1987; Eckert *et al.*, 1996; Thompson *et al.*, 2003; Wang *et al.*, 2005; Simón-Porcar *et al.*, 2015; Zhou *et al.*, 2015). The most extensively studied example of stylar polymorphism is heterostyly, in which differences in style and stamen height between the floral morphs result in reciprocal herkogamy, and this arrangement of reproductive organs promotes disassortative pollination (Darwin, 1877; Ganders, 1979; Barrett, 1992; Lloyd and Webb, 1992). A large body of empirical and theoretical work has investigated the repeated evolutionary breakdown of heterostyly, typically involving reduced herkogamy, biased morph ratios, the spread of homostylous variants, and increased selfing (Barrett, 1979, 1989; Charlesworth and Charlesworth, 1979; Belaoussoff and Shore, 1995; Vallejo-Marín and Barrett, 2009; Yuan *et al.*, 2023). These earlier findings provide a conceptual framework for understanding the maintenance and evolutionary breakdown of other stylar polymorphisms in angiosperms.

**Fig. 1.**
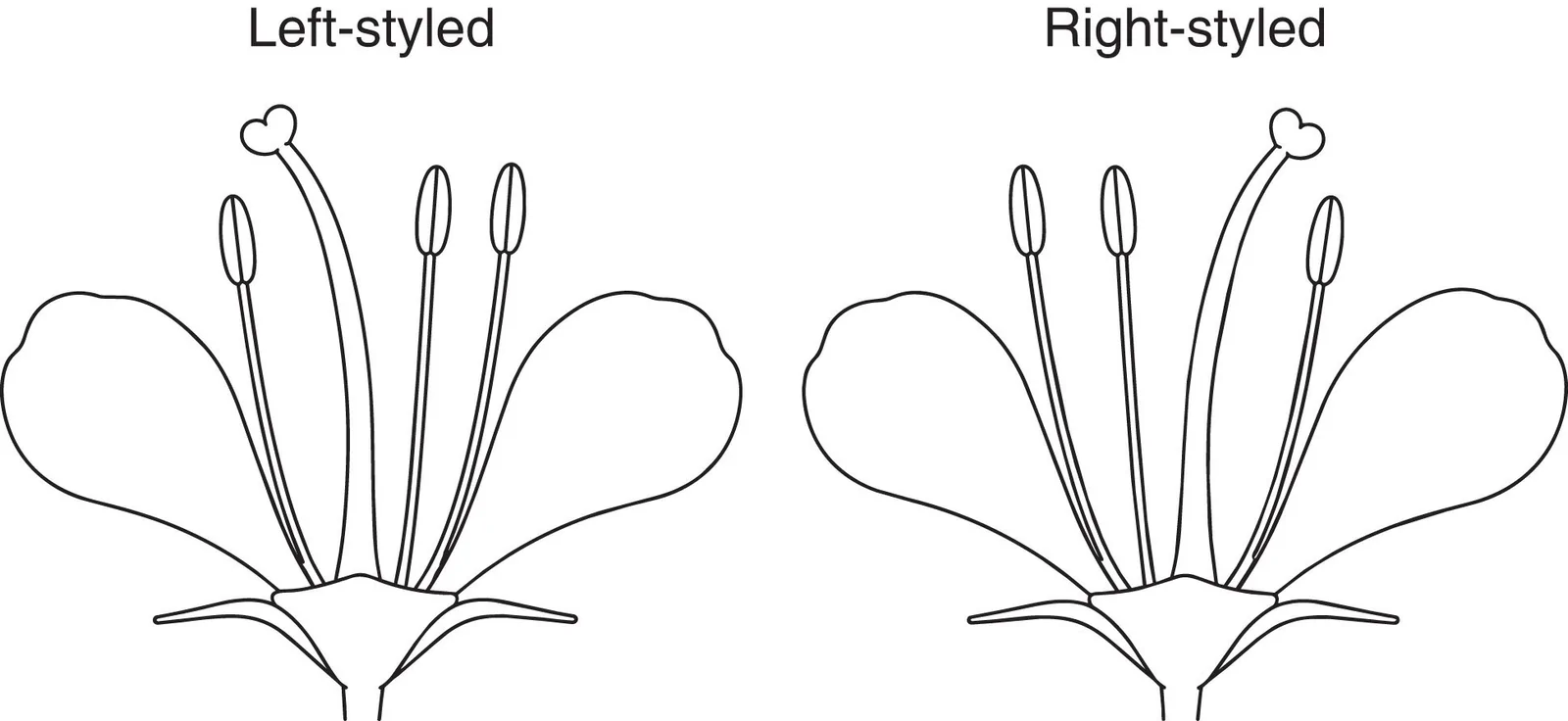
Diagram showing enantiostyly, with left-styled and right-styled flowers as mirror images of each other. Note that in *Wachendorfia* one anther is deflected to the same side as the style and two anthers are deflected to the other side. Illustrations were generated with assistance from ChatGPT (OpenAI).

Enantiostyly is a less well-known stylar polymorphism, in which plants possess styles deflected either to the left or right side of a flower and are therefore effectively mirror images of one another (Fig. 1; and see Ornduff and Dulberger, 1978; Barrett and Fairnie, 2024). Two fundamentally different types of enantiostyly occur in angiosperms: in monomorphic enantiostyly, flowers of both stylar orientations occur on the same plant, whereas in dimorphic enantiostyly, individual plants produce either left- or right-handed flowers, hereafter L- and R-plants (see Barrett, 2002: Fig. 2). Enantiostylous flowers exhibit pronounced reciprocal herkogamy, whereby the majority of pollen from the two morphs is spatially separated on opposite sides of pollinators such that most mating is expected to be disassortative, occurring between morphs (Jesson and Barrett, 2002*a*, *b*; Minnaar and Anderson, 2021). Disassortative mating between the two stylar morphs is expected to result in negative frequency-dependent selection (Barrett *et al.*, 2000; Thompson *et al.*, 2003), which should maintain equal morph ratios because rare morphs have increased mating opportunities until equilibrium is reached (much like selection for equal sex ratios in animals; Fisher, 1930). However, if mating becomes more random or even assortative (including selfing), the advantage of being rare diminishes and so too should the strength of negative frequency-dependent selection. Consequently, morphological traits that increase self-pollination or reduce disassortative mating (e.g., reduced herkogamy) should reduce negative frequency-dependent selection and lead to the breakdown of morph ratio equality. Although enantiostyly has evolved repeatedly across angiosperms (Jesson and Barrett, 2003) and shares several functional similarities with heterostyly, including populations with equal morph ratios (Jesson and Barrett, 2002*a*), much less is known about evolutionary modifications to this polymorphism. Reduced pollinator service in enantiostylous populations might make plants more reliant on self-pollination and the polymorphism more vulnerable to evolutionary breakdown (Jesson and Barrett, 2002*a*). However, empirical studies on the breakdown of enantiostyly and explicit tests of whether reduced herkogamy can lead to biased morph ratios and the emergence of homostylous forms (self-pollinating phenotypes with anthers and stigmas at the same position within a flower) are lacking.

**Fig. 2.**
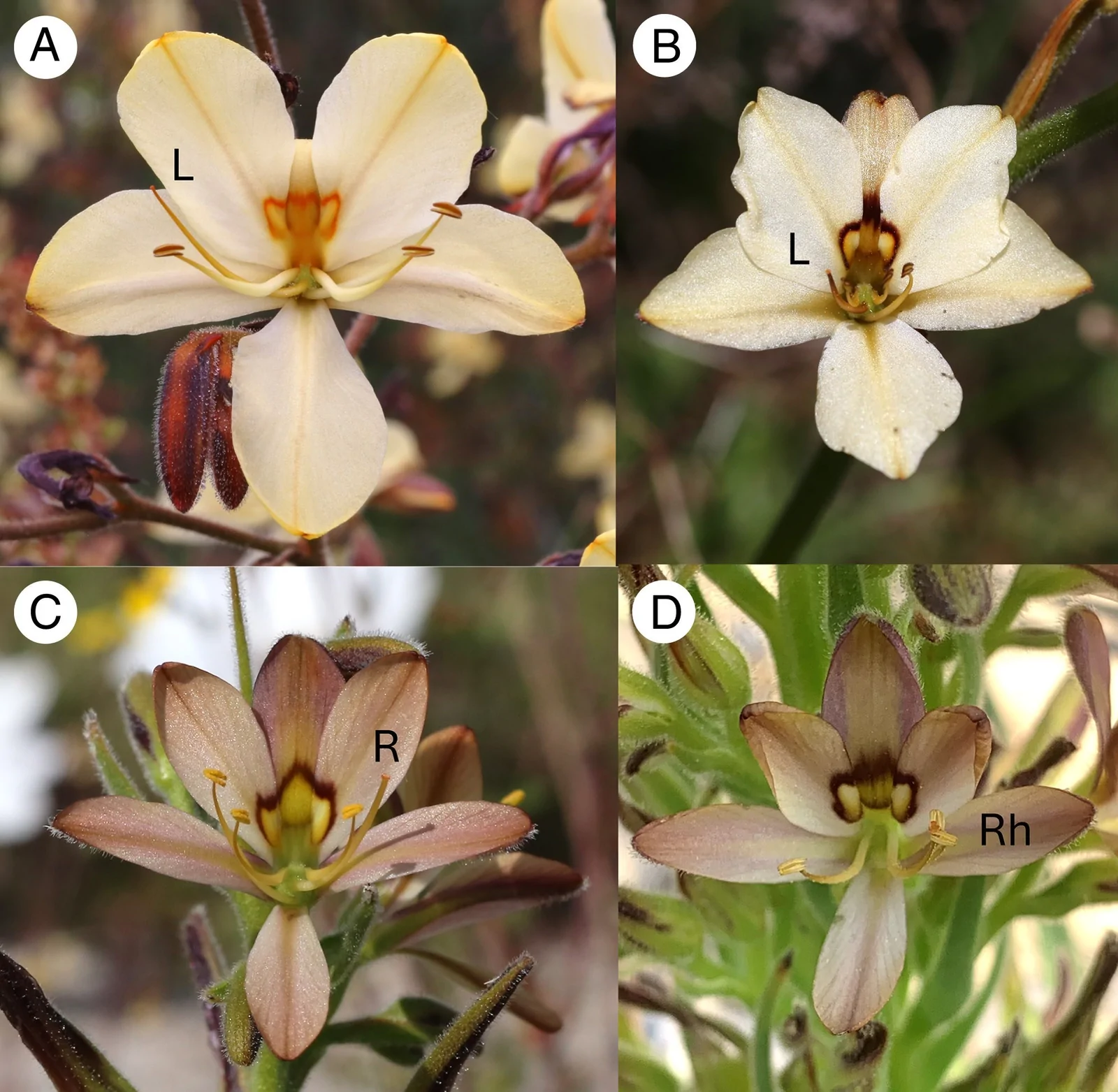
(A) Left-styled (L) flower of *Wachendofia paniculata* from Waylands. (B) Left-styled (L) flower of *W. brachyandra* from Pagasvlei. (C) Right-styled (R) flower of *W. multiflora* from Koeberg. (D) Right-styled homostylous (Rh) flower of *W. multiflora* from Redelingshuys with two anthers on the same side as the style.

The South African endemic genus *Wachendorfia* (Haemodoraceae) provides an opportunity to address this knowledge gap. All four species in the genus exhibit dimorphic enantiostyly and occur across a range of ecological contexts within the Cape Floristic Region, where pollinator service can be highly variable and pollinator limitation of seed set is not uncommon (Johnson and Bond, 1997; Rodger and Ellis, 2016). Although disassortative pollination has been demonstrated in a population of *W. paniculata* with equal morph ratios (Minnaar and Anderson, 2021), there is some evidence that *Wachendorfia* populations display variation in morph ratios and in the degree of spatial separation between reproductive organs (Jesson and Barrett, 2002*a*), suggesting the possibility of ongoing transitions in mating strategy. To test the hypothesis that enantiostyly in *Wachendorfia* might be subject to the same processes that have resulted in the evolutionary breakdown of heterostyly, we adopted a comparative approach across a total of 42 populations of three species: *W. paniculata*, *W. brachyandra* and *W. multiflora* (*= W. parviflora*) (Fig. 2). Note that we did not examine *W. thrysiflora*, the remaining species in the genus, which also possesses dimorphic enantiostyly. Populations of this species are highly clonal and most commonly monomorphic for style deflection (Jesson and Barrett, 2002*a*). Opportunities for evolutionary modifications to floral traits in *W. thrysiflora* are likely to be limited because of the low levels of reproduction through seed in most populations.

Our study addressed the following specific questions.

1. Are biased morph ratios of the three *Wachendorfia* species and the occurrence of homostylous variants more characteristic of populations with reduced stigma–anther separation?
2. Is pollinator service more reliable in populations with equal morph ratios and greater herkogamy?
3. Is the lower degree of herkogamy reported for *W. multiflora*, relative to *W. paniculata* (Jesson and Barrett, 2002*a*), associated with reduced disassortative pollination and increased self-pollination?
4. What are the levels of self-compatibility in each of the three species and how reliant are they on autonomous self-pollination for seed set?

## MATERIALS AND METHODS

### Style morph ratios and floral traits

To determine the relationships between floral morph frequencies and floral traits, we sampled 15 populations of *Wachendorfia multiflora* (Klatt) J.C.Manning & Goldblatt, 17 populations of *W. paniculata* Burm. (including forms 1 and 3; for a description of these forms, see Helme and Linder, 1992) and ten populations of *W. brachyandra* W.F.Barker (Supplementary Data Table S1). Note that we did not include any populations of *W. paniculata* form 2, which is known only from the Cederberg region. The species flower during spring (August–October) and are distributed across the South African Cape region, where they often occur in open fynbos vegetation, which is prone to fire. For each population, we determined the number of left- and right-handed individuals (from the perspective of the onlooker) from a minimum of 15, but ≤828 individuals (Supplementary Data Table S1), depending on population size. We tested whether the morph ratio differed from 1:1 using one-proportion *z*-tests and calculated bias as the deviation from equal morph ratios using the formula:


$$\mathrm{bias}=\left|0.5-\left(\frac{\mathrm{number}\;\mathrm{of}\;\mathrm{left}\;\mathrm{plants}}{\mathrm{number}\;\mathrm{of}\;\mathrm{left}\;\mathrm{plants}\;+\;\mathrm{number}\;\mathrm{of}\;\mathrm{right}\;\mathrm{plants}}\right)\right|$$

In each population, we measured herkogamy as the distance between the stigma and closest anther (*n* = 42 populations), in addition to flower width (*n* = 41), which we use as a proxy of overall flower size. For some of the populations, we also measured several other traits, including the distance between the lower anthers (*n* = 26), distance between the two anthers on the same side of the flower (*n* = 35), distance between the stigma and the upper anther (*n* = 14) (for an illustration of these measurements, see Supplementary Data Fig. S1), daily floral display size, i.e. number of flowers open each day (*n* = 21), and estimated flowering population size (*n* = 41) (Supplementary Data Table S1). These additional measurements provided information on potential floral correlates of morph-ratio variation. We measured all traits using freshly opened flowers before noon, and a single randomly chosen flower per plant was sampled. We calculated mean values for each trait per population, which were then used in further statistical analyses. One of the data points, for the *W. multiflora* population at Red Hill, was adapted from the study by Jesson and Barrett (2002*a*).

In our statistical analysis, we used correlation tests to examine the relationships between herkogamy and the other measured floral traits. Given that the traits were correlated, we modelled them separately in R (R Core Team, 2025) to avoid issues of multicollinearity and to allow direct comparison of alternative, biologically motivated predictors. Population size was mean-centred and scaled to improve numerical stability during model fitting, whereas other traits were left unscaled because their natural measurement ranges did not pose convergence issues. Given that several predictor variables contained missing values, and complete-case analysis would have reduced sample sizes substantially, we applied multiple imputation (number of imputations *m* = 20) using predictive mean matching from the mice package (van Buuren and Groothuis-Oudshoorn, 2011), allowing all predictors except the response variable (morph bias) to be imputed. For each imputed dataset, we fitted a beta regression using glmmTMB (Brooks *et al.*, 2017) with species as random factor, then combined parameter estimates across imputations using Rubin’s rules (Rubin, 1988). To identify the best predictor of morph-ratio bias, we calculated the Akaike information criterion (AIC) for all candidate models. We also included a null model containing only the intercept and random effect to provide a baseline for AIC comparisons. Using the predictor from the model with the best fit (herkogamy, see Results), we also fitted beta regressions for each of the three species separately and extracted their slopes. Additionally, we examined the likelihood of homostylous variants occurring in populations (1 = present, 0 = absent) in response to herkogamy using a binomial general linear model (GLM).

### Pollinator observations

To investigate whether differences in the pollination environment might be associated with contrasting morph frequencies between populations, we carried out a total of 30 h of pollinator observations (Supplementary Data Table S2) spread over 16 populations (3 *W. brachyandra*, 7 *W. paniculata* and 6 *W. multiflora*) spanning a range of morph ratios. We recorded pollinator visits at only a subset of the 42 populations included in this study, resulting in differences in observation effort between the species (2.5 h for *W. brachyandra*, 9.6 h for *W. paniculata* and 17.9 h for *W. multiflora*). For each population, we identified insect visitors during spring (August–October) and determined their visitation rates. In each population, we conducted observations of a focal patch on days with favourable weather, without rain, after 09:00 but before 16:00 h, with the observer >1 m distance from the patch. Where we recorded repeated observations for a population, we used the mean visitation rate in further analyses. As indicated above, we imputed missing values, then used a beta regression to model morph ratio bias in response to visitation rate with species as a random factor and combined parameter estimates across imputations. Additionally, we determined the AIC value and compared it with the floral measurements above.

### Pollen transfer

To understand the role of disassortative pollination in maintaining equal morph ratios, we conducted field observations and pollen-dispersal experiments at three populations of *W. multiflora*. We focused on this species because it exhibited the most continuous variation in both herkogamy and morph-ratio bias. The three populations chosen displayed an equal morph ratio (Langebaan), mild bias (Koeberg) and strong bias (Redelingshuys). To measure both between- and within-plant pollen movement in these populations, we used the quantum dot protocol described by Minnaar and Anderson (2019).

For between-plant pollen movement, we marked anthers in three flowers on six focal left-handed plants each on two days, and the same number of right-handed plants on two different days. Each anther position received a distinct colour of quantum dots, which was identical in all other marked flowers and individuals on the same day. After marking flowers, we waited until the late afternoon to allow for pollinator visitation, then collected the styles of unmarked flowers surrounding the marked individuals. In the field, we mounted styles on microscope slides using coverslips and sticky tape. In the laboratory, we recorded the number of marked pollen grains from each quantum dot colour on each stigma using an Olympus CH20BIMF200 compound microscope at ×40 magnification and a hand-held Zartek ultraviolet torch. From these data, we calculated the percentage of pollen from each of the three anther positions that had reached stigmas of the same or of a different morph.

For within-plant pollen movement, we marked anthers of focal individuals in the morning, immediately after anthesis, with quantum dots as above. However, in this experiment all anthers from one individual were marked with the same colour, and each individual was marked with a different colour. Given that we had four colours available and repeated the experiment for two days per site, we collected data from a total of eight individuals per population. Styles of the marked individuals were collected in the late afternoon and mounted on microscope slides. The total number of pollen grains on each stigma was quantified as above, in addition to the number of pollen grains marked with quantum dots. Using GLMs with quasi-Poisson error structure in R followed by Tukey’s *post hoc* tests, we compared the total pollen deposition and the self-pollen deposition between the three sites. For the Koeberg and Redelingshuys populations, we also measured herkogamy of each marked flower immediately before collecting the stigma and measured the amount of self-pollen deposited. Using these data from individual plants, we modelled the deposition of self-pollen on stigmas in relationship to herkogamy using a GLM with negative binomial error structure in R and site as a random factor.

### Pollination experiments

To determine whether each of the three study species can produce selfed seeds, either autonomously or through manual self-pollination, and the extent to which seed set in populations might be pollen limited, we conducted experimental hand pollinations in the field for a single population of each species. Experiments were carried out on *W. paniculata* at Camps Bay (form 1) during October 2022, *W. brachyandra* at Kenilworth Racecourse during October 2022, and *W. multiflora* at Langebaan during August 2023. Because many of the seeds resulting from hand pollinations were lost at Langebaan, a second season was necessary to achieve an adequate sample size for statistical analysis. However, in 2024 there was severe porcupine herbivory of plants at the Langebaan site, thus we had to carry out the experiments at a second morphologically similar population of the species located 5 km from the Langebaan site in the Postberg section of the West Coast National Park.

At each population, we covered flowers with mesh bags in the morning before anthesis, marked them with cotton string and applied the following six treatments by hand using forceps wiped clean between treatments: (1) autonomous self-pollination, where flowers were left undisturbed inside mesh bags; (2) self-pollination, where stigmas were saturated with pollen from flowers of the same plant; (3) intramorph cross-pollination, where pollen from another individual of the same morph was placed on stigmas; (4) intermorph cross-pollination, where pollen from another individual of a different morph was placed on stigmas; (5) anther removal and open pollination, where mesh bags were removed and anthers cut off; and (6) open pollinated, where mesh bags were removed and flowers were left undisturbed to allow visitation by pollinators. We collected fruits 2–3 weeks later, once seeds had developed, and the number of seeds per fruit (range zero to three) was determined. We used GLMs with Poisson error distribution in R for each of the three species separately, with plant ID as a random factor, followed by Tukey’s *post hoc* tests. For *W. multiflora*, we combined the data collected in 2023 and 2024 and included site as a nested random factor in the model.

## RESULTS

### Relationship between morph ratios and floral traits

**Fig. 3.**
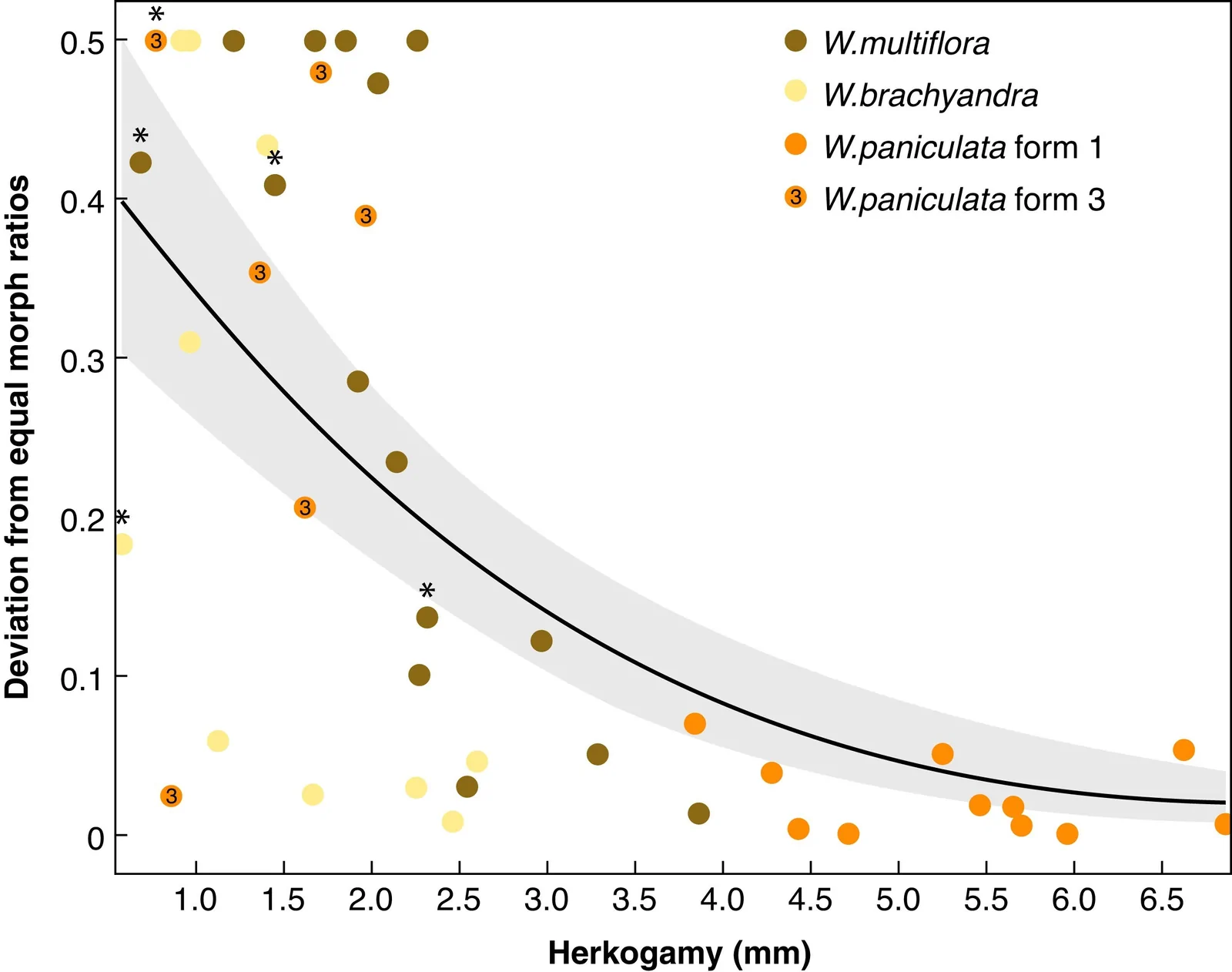
The relationship between deviation from equal morph ratios and herkogamy in 42 populations of three species of *Wachendorfia* [*W. multiflora*, brown; *W. brachyandra*, yellow; *W. paniculata*, orange (unfilled, form 1; 3 indicates form 3)]. A deviation of zero represents equal morph ratios and of 0.5 populations with only a single morph. An asterisk above a dot indicates the presence of homostylous variants in a population. The grey shaded area represents the confidence interval predicted by a beta regression with species as a random factor.

The relative frequencies of L- and R-plants showed considerable variation in the three species of *Wachendorfia* (Fig. 3; Supplementary Data Table S1), ranging from populations where the ratio of morphs was not significantly different from 1:1 (*n* = 21 populations) to populations fixed for a single morph (*n* = 7 populations). In addition, homostylous variants, in which two stamens were deflected to the same side as the style (Fig. 2D), were found in five populations (*W. paniculata* at Pearly Beach, *W. multiflora* at Redelingshuys, Banghoek and Donkie’s Kraal, and *W. brachyandra* at Rondebosch Common). Populations of *W. paniculata* form 1, which is the most common and widespread form (see Helme and Linder, 1992), exhibited morph ratios close to equality and larger values of herkogamy. In contrast, form 3, which is characterized by its reduced height and narrow, hairy leaves and grows along the southwest coast of the Cape, where it can occur in sympatry with form 1 (Helme and Linder, 1992), exhibited reduced herkogamy and often pronounced morph biases. Likewise, morph-ratio biases occurred frequently in *W. brachyandra* populations and were most evident in *W. multiflora* populations.

Reduced anther–stigma separation within flowers was significantly associated with increased bias in morph ratio across our total sample of populations (Fig. 3). Populations with pronounced herkogamy were more likely to have equal morph ratios, whereas those with reduced herkogamy often exhibited biased morph ratios or were fixed for one morph. Pseudo-*R*^2^ ranged from 0.39 in *W. brachyandra* to 0.55 and 0.56 in *W*. *multiflora* and *W. paniculata*, respectively, indicating moderate to strong explanatory power for these relationships. All three species also showed significant relationships between morph bias and herkogamy independently, although their slopes differed owing to their differences in range (*W. paniculata* β = −0.51 ± 0.14 s.e., *P* < 0.001; *W. brachyandra* β = −1.11 ± 0.43 s.e., *P* = 0.010; *W. multiflora* β = −1.05 ± 0.25 s.e., *P* < 0.001). Furthermore, in populations with pronounced herkogamy, we observed no homostylous variants, but when herkogamy decreased below 2.3 mm the probability of encountering homostylous variants increased exponentially (χ^2^ = 8.26, d.f. = 41, *P* = 0.004), consistent with a threshold-like transition rather than a gradual linear relationship.

In addition to herkogamy, flower width, distance between the lower anthers, distance between the side anthers and population size each showed a significant relationship with morph ratio (Table 1). As each of these trait measurements decreased, the likelihood of biased morph ratios increased. We detected no relationship between morph ratio and daily display size or the distance between the upper anther and the stigma (Table 1). Model comparison indicated that herkogamy was the strongest predictor, because the model including this trait had the lowest AIC value. Flower width showed moderate support, whereas all other models received little support (Table 1). Herkogamy had positive relationships with all other traits measured but was most strongly correlated with flower width (*ρ* = 0.85, *P* < 0.001).

**Table 1.** Model output and Akaike information criterion for beta regression models predicting morph bias in *Wachendorfia* populations in response to different floral traits, with species as a random factor. Significant results are given in bold.

Trait	Estimate	s.e.	*t*	*P*-value	AIC	ΔAIC	Support
**Herkogamy**	**0**.**62**	**0**.**11**	**5**.**74**	**<0**.**001**	**−119**.**39**	**0**.**0**	**Best**
**Flower width**	**0**.**11**	**0**.**02**	**5**.**01**	**<0**.**001**	**−115**.**89**	**3**.**5**	**Moderate**
**Lower anthers**	**0**.**17**	**0**.**04**	**3**.**87**	**<0**.**001**	**−106**.**69**	**12**.**7**	**Very weak**
Display size	0.21	0.23	0.91	0.371	−96.41	23.0	Very weak
**Side anthers**	**0**.**30**	**0**.**14**	**2**.**06**	**0**.**041**	**−96**.**17**	**23**.**2**	**Very weak**
**Scaled population size**	**0**.**45**	**0**.**22**	**2**.**08**	**0**.**038**	**−95**.**69**	**23**.**7**	**Very weak**
Upper distance	0.06	0.06	0.95	0.347	−94.42	25.0	Very weak
Insect visitation rate	0.15	0.10	1.41	0.235	−93.14	26.3	Very weak
**Null model**	**1**.**52**	**0**.**38**	**3**.**99**	**<0**.**001**	**−92**.**30**	**27**.**1**	**Very weak**

### Pollinator observations

**Fig. 4.**
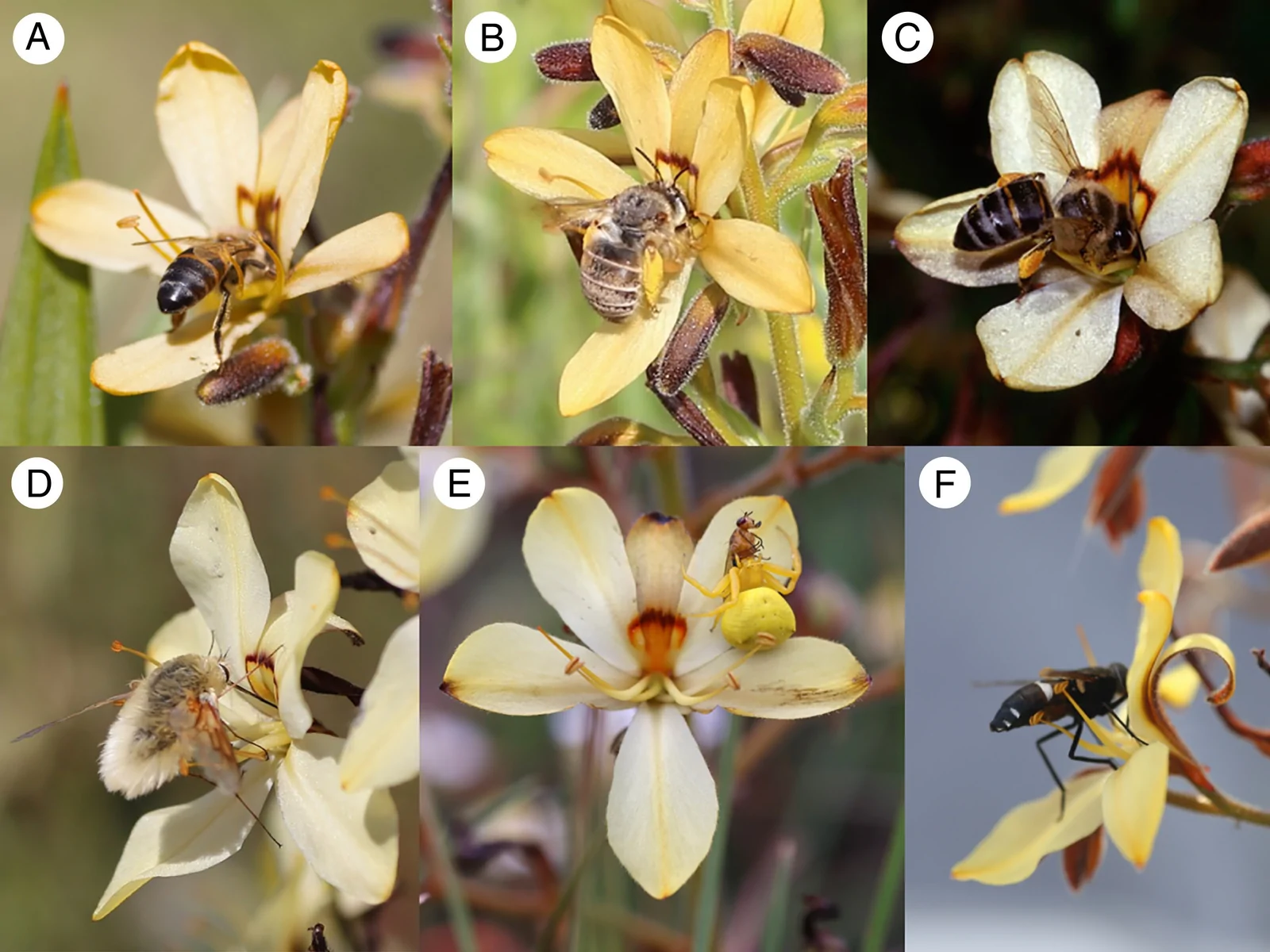
Arthropod visitors of *Wachendorfia* study species. (A) *Apis mellifera* on *W. multiflora*. (B) *Anthophora wartmanni* on *W. multiflora*. (C) *Apis mellifera* on *W. brachyandra* (photograph by Colin Paterson-Jones ©). (D) *Australoechus hypoleucus* on *W. paniculata*. (E) Thomisid spider with unidentified fly on *W. paniculata*. (F) *Philoliche angulata* on *W. paniculata*.

Honeybees (*Apis mellifera*) were the most common flower visitors to *Wachendorfia* species and were observed at 9 of the 16 populations surveyed (Supplementary Data Table S2; Fig. 4). At three of the populations, we did not observe any floral visitors, and at the remaining populations we observed visits only from other species. These included solitary bee species, which were recorded at five of the surveyed populations, such as carpenter bees (*Xylocopa afra*), *Amegilla* sp., *Anthophora wartmanni*, *Rediviva peringueyi*, *Rediviva parva* and two unidentified smaller species. Furthermore, we observed several fly visitors, such as the long-proboscid fly *Philoliche angulata*, syrphid flies and *Australoechus hypoleucus*. We also observed asilid flies, an unidentified fly species, hopliinid beetles and thomisid spiders on flowers, but discounted these species as reliable pollinators owing to the importance of wing-pollination in Haemodoraceae (see Minnaar and Anderson, 2021; Johnson *et al.*, 2023, 2026). Visitation rates during the observation periods varied widely between populations, ranging from zero insect visitors at three sites to 7.70 visits per flower h^−1^ at the *W. multiflora* population at Capri. The anthers of flowers typically contacted the wings of visitors in *W. paniculata* and *W. multiflora*, whereas in *W. brachyandra* we observed that the lower anthers often made contact with the abdomen of honeybees, but the stigma and upper anther often contacted the front legs or corbicula.

In contrast to our predictions, and despite the considerable variation in visitation rates among populations, we found no evidence that populations with greater morph-ratio bias and reduced herkogamy received fewer insect visits. Visitation rate showed no significant relationship to morph-ratio bias, and the corresponding model had the highest AIC, indicating the weakest support (Table 1).

### Pollen transfer

**Fig. 5.**
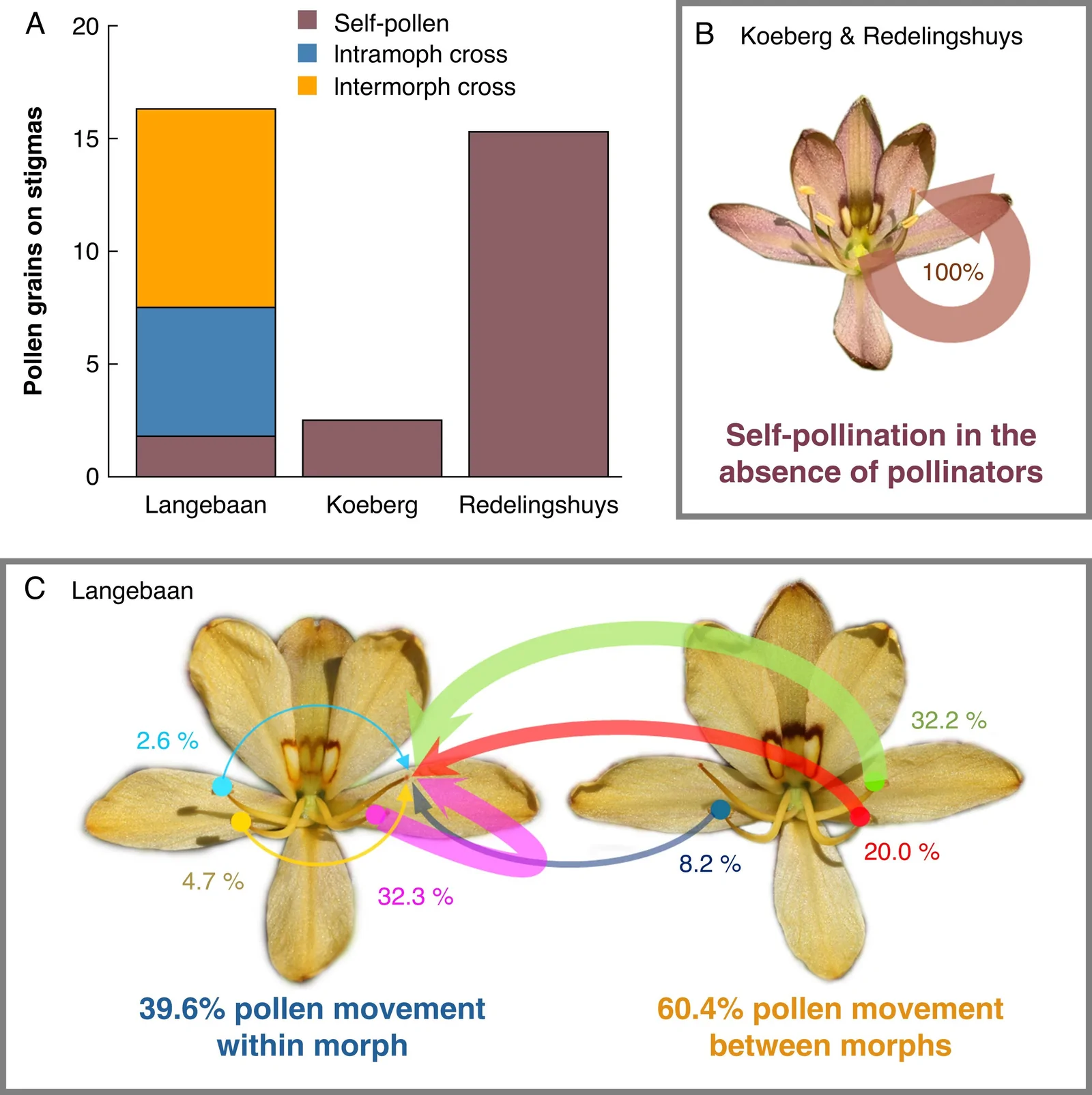
(A) Number of pollen grains on stigmas of *Wachendorfia multiflora* in the Langebaan population (pronounced herkogamy and frequent visitation), Koeberg population (pronounced herkogamy and no visitation) and Redelingshuys population (low herkogamy and no visitation). (B) Self-pollination within a flower; image from the Redelingshuys population 2025. Note reduced flower size and herkogamy. (C) Cross-pollination between plants of the same and different morph; image from the Langebaan population 2023.

We predicted that our quantum-dot analysis of pollen dispersal in *W. multiflora* would reveal weaker disassortative pollen transfer and increased self-pollination relative to previously published results for form 1 of *W. paniculata* (see Minnaar and Anderson, 2021), which displays a greater degree of herkogamy. This prediction was confirmed. At the Langebaan population of *W. multiflora* (*n* = 513 stigmas), we found ∼60 % of cross-pollen was dispersed between morphs by pollinators (Fig. 5C), in comparison to Minnaar and Anderson (2021), who reported ∼85 % cross-pollen movement between morphs. Because no flower visits were observed at the Redelingshuys and Koeberg populations and all pollen found on their stigmas could be attributed to within-flower selfing, we did not collect stigmas for inter-plant pollen movement at these populations.

At the Langebaan population, which received frequent pollinator visits (1.55–3.12 visits per flower h^−1^), we recorded 11 % self-pollen deposited on stigmas, a value similar to that reported by Minnaar and Anderson (2021) for *W. paniculata* (8 %). In contrast, the two *W. multiflora* populations in which we observed no pollinator visits during our study period, as might be predicted, received 100 % self-pollen. Average total (χ^2^ = 16.55, d.f. = 2, *P* < 0.001) and self-pollen loads (χ^2^ = 30.69, d.f. = 2, *P* < 0.001) varied significantly among the three *W. multiflora* populations (Table 2; Fig. 5A). Total pollen loads were similar at Langebaan and Redelingshuys (*z* = 0.24, *P* = 0.968), whereas Koeberg had significantly less total pollen deposition than the other two populations (*z* = 3.05, *P* = 0.006). The Koeberg and Langebaan populations showed similar degrees of herkogamy and received equivalent amounts of self-pollen (*z* = 0.55, *P* = 0.847), whereas Redelingshuys, with smaller herkogamy values, received more self-pollen than both Koeberg (*z* = 3.15, *P* = 0.005) and Langebaan (*z* = 3.38, *P* = 0.002). Notably, the average total pollen load (2.5 grains per stigma) at the Koeberg population was almost as low as self-pollen deposition in the Langebaan population (1.9 grains per stigma from autonomous and geitonogamous pollen transfer).

**Table 2.** Observed morph bias, mean herkogamy, visitation rate (in visits per flower per hour), mean number of pollen grains deposited on stigmas, percentage of outcross pollen dispersed within and between morphs and the percentage of pollen received from self-pollination for three populations of *Wachendorfia multiflora*.

Population	Morph bias	Herkogamy (mm)	Visitation rate (visits per flower h^−1^)	Total pollen (grains per stigma)	self-pollen (%)	Intermorph cross (%)	Intramorph cross (%)
Langebaan	0.05	3.3	2.34	16.3	11	60.4	39.6
Koeberg	0.12	3.0	<0.01	2.5	100	–	–
Redelingshuys	0.42	0.7	<0.01	15.3	100	–	–

**Fig. 6.**
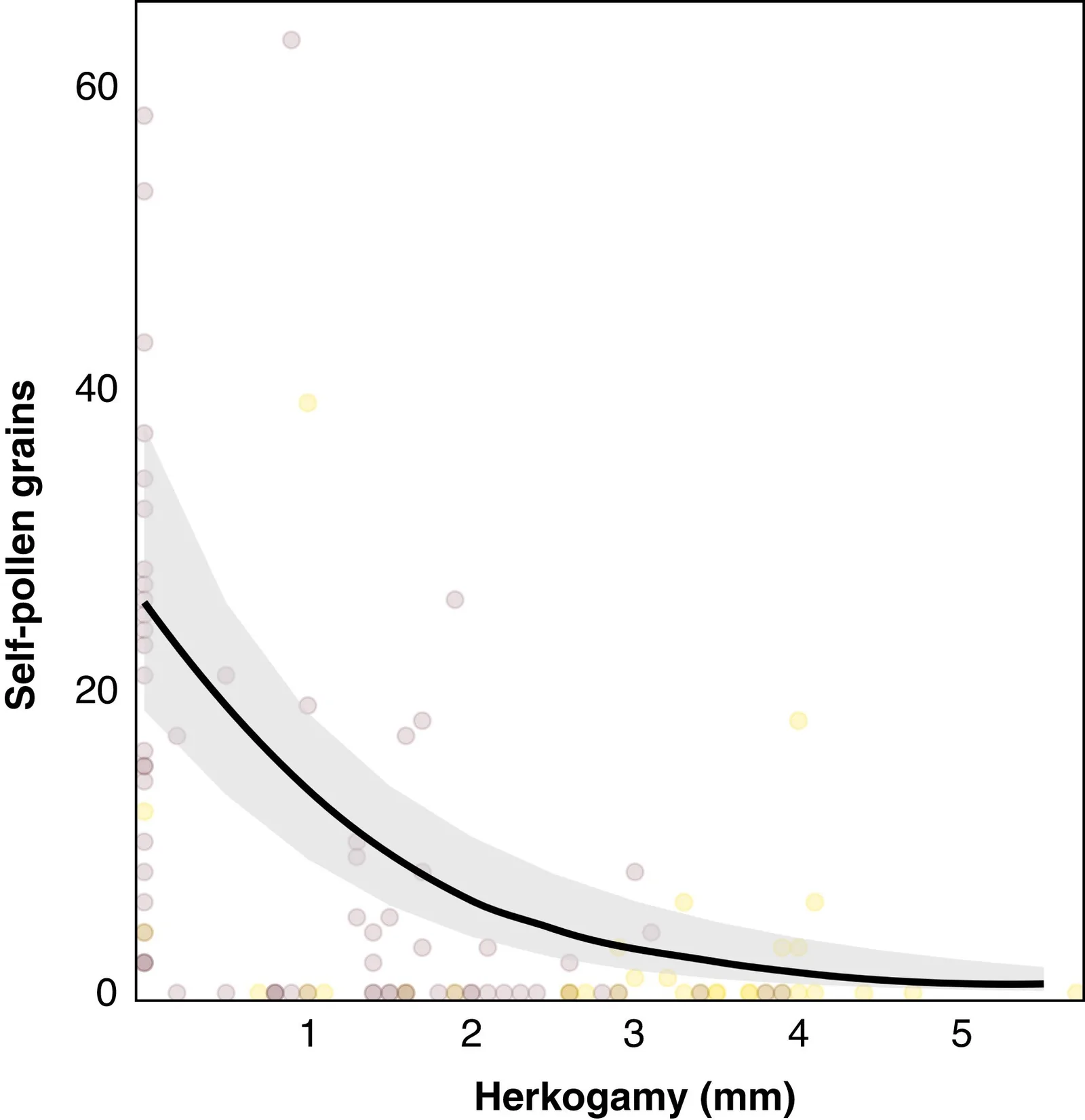
The relationship between self-pollen deposition on stigmas and the degree of herkogamy in the Koeberg and Redelingshuys populations of *Wachendorfia multiflora*, in which no pollinator visits were observed in 2025. Each point represents measurements of self-pollen deposition and herkogamy values from a marked flower. Flowers from Redelingshuys in brown (*n* = 74) and flowers from Koeberg in yellow (*n* = 33). The grey shaded area represents the confidence interval predicted by a GLM with negative binomial error structure and site as a random factor.

To investigate whether the amount of self-pollen on stigmas is associated with the degree of herkogamy, we pooled data for individual flowers from the Koeberg and Redelingshuys populations where no insect visitors were recorded and quantum dot analysis indicated that 100 % self-pollen deposition. As might be predicted, a significant association between the two variables was observed, with smaller herkogamy values associated with larger amounts of self-pollen deposition (Fig. 6; χ^2^ = 34.5, *P* < 0.001).

### Pollination experiments

**Fig. 7.**
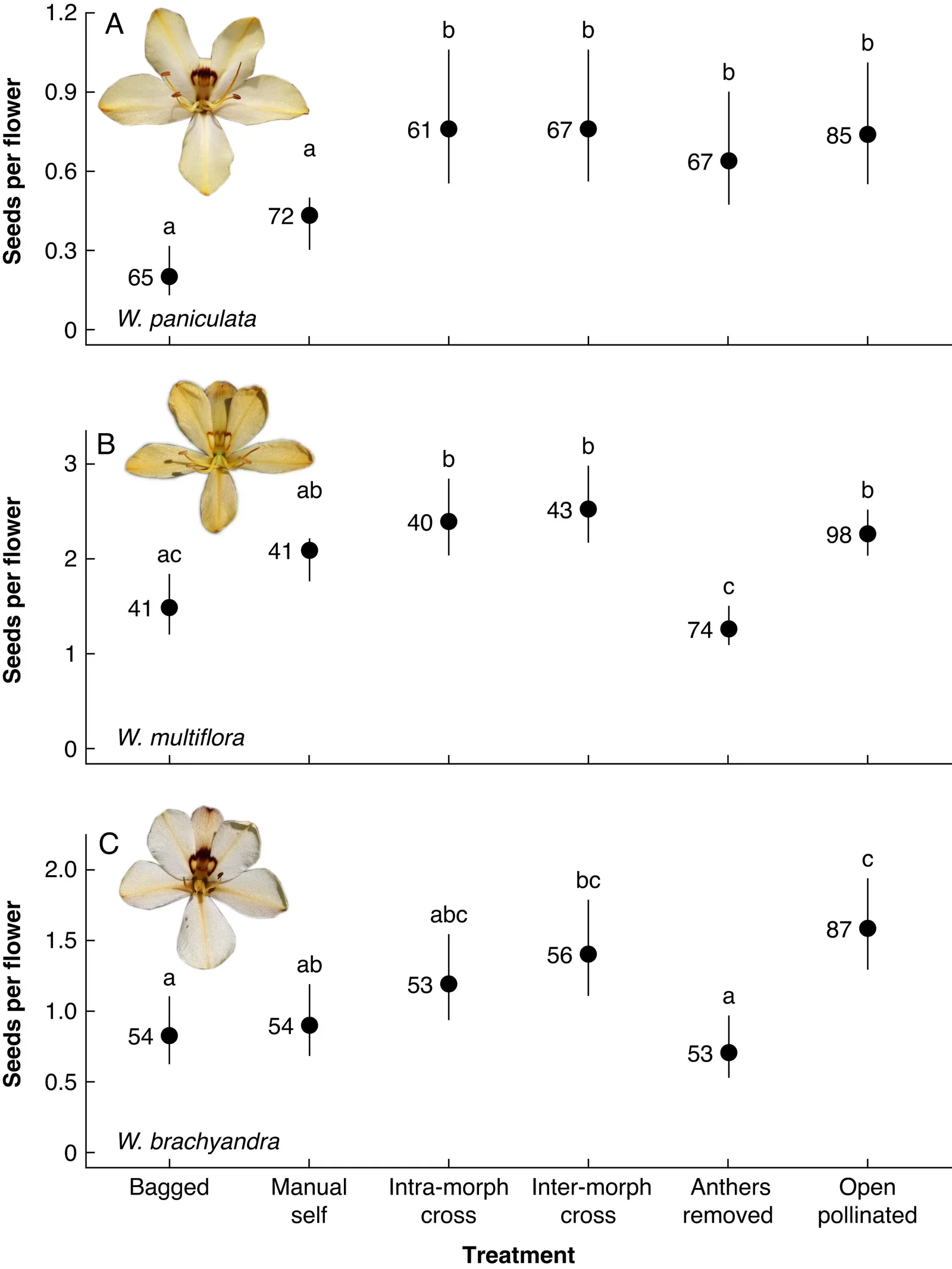
Seed set from experimental self- and cross-pollinations of three species of *Wachendorfia*. The number of seeds produced per flower following the various treatments are indicated. (A) *W. paniculata*, Camps Bay form 1. (B) *W. multiflora*, Langebaan and West Coast National Park combined. (C) *W. brachyandra*, Kenilworth Racecourse. Error bars are 95 % confidence intervals. Sample sizes (number of flowers) are shown next to the mean for each treatment. Mean seed set values that share letters are not significantly different based on GLM analyses with Poisson distribution and plant ID as random factor. Flower images are presented at the same scale.

In all three *Wachendorfia* species, seed set differed among the six experimental treatments performed (*W. paniculata*: χ^2^ = 39.03, d.f. = 5, *P* < 0.001; *W. multiflora*: χ^2^ = 37.16, d.f. = 5, *P* < 0.001; *W. brachyandra*: χ^2^ = 38.25, d.f. = 5, *P* < 0.001), but not between mophs. Seed set was also not significantly different between intermorph and intramorph cross-pollinations for any of the three species. Furthermore, there was no evidence that plants were pollen limited based on a comparison of seed set in open-pollinated versus cross-pollinated flowers (Fig. 7; Supplementary Data Table S3). This was supported by low indices of pollen limitation (1 − intermorph cross/open pollination) which was ≤0.1 in all three species, where values close to zero are associated with no pollen limitation.

In *W. paniculata*, seed set from bagged flowers and manually self-pollinated flowers did not differ significantly, but both treatments produced significantly fewer seeds than all other treatments (Fig. 7A), consistent with the presence of a weak self-incompatibility system and/or post-zygotic inbreeding depression in *W. paniculata*. In contrast, seed set from manual self- and cross-pollination did not differ significantly in either *W. multiflora* (Fig. 7B) or *W. brachyandra* (Fig. 7C), indicating a higher degree of self-compatibility and the absence of a self-incompatibility system in these species. This was also reflected by differences in the index for pollinator contribution to seed set (1 − autonomous self-pollination/open pollination): *W. paniculata* displayed a relatively high value of 0.73 (close to one), suggestive of a high pollinator contribution to seed set; in comparison, *W. multiflora* and *W. brachyandra* had values of 0.34 and 0.48, respectively, suggestive of lower pollinator contributions (values of zero indicate a total lack of pollinator contribution).

No significant difference in seed set was detected between open-pollinated flowers and flowers with their anthers removed in *W. paniculata*, suggesting that a large component of pollen receipt is from other flowers. In contrast, seed set was significantly higher in open-pollinated flowers of both *W. multiflora* and *W. brachyandra* in comparison to flowers without anthers (Fig. 7), as expected if these species obtain much of their pollen through self-pollination. This finding was also reflected by differences in the indices of self-contribution (1 − anthers removed/open pollination), with the lowest value of 0.15 in *W. paniculata* (a value of zero indicates no self-contribution), and higher self-contribution of 0.44 and 0.55 in *W. multiflora* and *W. brachyandra*, respectively (values of one indicate completely selfing).

## DISCUSSION

Studies of diverse heterostylous species have demonstrated that reduced herkogamy, biased morph ratios and the sporadic emergence of homostylous variants often signal the breakdown of floral polymorphism and transitions to self-fertilization (reviewed by Ganders, 1979; Barrett, 2019). Here, we investigated whether this well-known mating-system transition can be extended to a different class of stylar polymorphism: enantiostyly. We found that there was a strong exponential relationship between variation in herkogamy and morph ratios (Fig. 3). A reduced distance between stigmas and anthers was significantly associated with unequal morph ratios and an increased likelihood of morph fixation. This pattern was observed in populations of all three enantiostylous species and was more pronounced when data were pooled to generate a wider range of herkogamy values. Furthermore, we found that across several *W. multiflora* populations, reduced herkogamy was associated with increased amounts of self-pollen on stigmas. Finally, we also observed sporadic homostyles in five populations, across all three species, and that these occurred more frequently in populations with reduced herkogamy and skewed morph ratios. These findings provide a mechanistic link between reduced herkogamy and self-pollination, with implications for the breakdown of stylar polymorphisms.

### Selfing and assortative mating in *Wachendorfia*

One of the best predictors of self-pollination in angiosperms is the degree of herkogamy: the closer the reproductive parts are within a flower, the more likely self-pollination is to occur (reviewed by Opedal, 2018). Enantiostylous flowers in *Wachendorfia* reduce the amount of self-pollination by separating anther and stigmatic positions such that pollen from two of the three anthers is placed on the opposite side of visiting pollinators to the site of pollen receipt. Minnaar and Anderson (2021) demonstrated that pollen from anthers on the left side of flowers very rarely reaches right-hand stigmas and vice versa, making the presence of both morphs within populations crucial for efficient cross-pollen transfer. However, there are ecological situations where pollen movement between different morphs might become compromised, increasing the likelihood of self-pollination and/or assortative pollen transfer. For example, the colonization of new sites can often be associated with small populations, and this can lead to skewed morph ratios owing to genetic drift and founder effects (Barrett *et al.*, 1989). Low population densities can also make pollinator movement more stochastic and decrease transitions between plants of different morphs (Saltini *et al.*, 2025), thus resulting in increased self-pollination. These demographic factors seem likely to influence the efficacy of cross-pollination in our focal species.

*Wachendorfia* populations tend to be more sparsely distributed in dry habitats, potentially affecting cross-pollination and thus mating patterns. Low visitation rates by pollinators often result from low population densities, small population size (Ågren *et al.*, 2008; Courtice *et al.*, 2020) or competition with other rewarding plants (Kemp *et al.*, 2022). When pollen movement between morphs is unreliable, especially in populations with skewed morph ratios, assortative cross-pollination might be advantageous in *Wachendorfia*. This is possible because, in contrast to most heterostylous species, which possess heteromorphic incompatibility (Ganders, 1979; Barrett, 1992), assortative pollination in *Wachendorfia* is fully compatible, as demonstrated in our controlled pollination study (Fig. 7; also see Jesson and Barrett, 2002*a*). Moreover, in *Wachendorfia* species, one of the three anthers is deflected to the same side of the flower as the stigma, facilitating pollen transfer within a morph through assortative pollination. In our study of *W. multiflora* using quantum dots, we found that this anther promoted significant pollen transfer between plants of the same morph, a result also found in *W. paniculata* (Minnaar and Anderson, 2021).

Herkogamy generally limits interference between reproductive organs and thus reduces the incidence of self-pollination (Webb and Lloyd, 1986). However, we have outlined above ecological conditions that might promote the evolution of self-pollination through a reduction in the physical distance separating anthers and stigmas. Autonomous self-pollination might potentially provide reproductive assurance in these ecological circumstances (Busch and Delph, 2012). Significantly, in *W. multiflora* we observed low self-pollination in flowers with strong herkogamy and that self-pollen on stigmas increased exponentially as the distance between anthers and stigmas decreased (Fig. 6). We also found that some populations of *W. multiflora* received no pollinator visits during our observation periods and appeared to rely on self-pollination for seed production, consistent with the reproductive assurance hypothesis.

In numerous angiosperm species, the increased incidence of self-pollination resulting from a reduction in herkogamy is associated with modifications to other floral traits (e.g. Lloyd, 1965; Morgan *et al.*, 1989). Although reduced herkogamy provides the direct proximate floral mechanism causing self-pollen deposition on stigmas, if high selfing rates persist over generations, other changes to reproductive traits often evolve in association. These include smaller flower sizes, less intense floral colours, reduced nectar volumes and a decrease in pollen production. These correlated changes associated with mating-system transitions frequently give rise to the evolution of the selfing syndrome (reviewed by Sicard and Lenhard, 2011). We did not measure all these floral traits in *Wachendorfia* but found that reduced herkogamy was significantly associated with smaller flower sizes, consistent with the early stages of the evolution of the selfing syndrome. This modification was especially evident in *W. multiflora*, *W. brachyandra* and form 3 of *W*. *paniculata* (also see Jesson and Barrett, 2002*a*).

Another consequence of persistent selfing in populations is purging of the genetic load, resulting in a reduction in the magnitude of inbreeding depression (Barrett and Charlesworth, 1991; Charlesworth and Willis, 2009). In our pollination study, *W. paniculata* was the only species to exhibit significantly higher seed set in experimental cross- versus self-pollinations (Fig. 7), a result consistent with earlier pollination studies of this species (Ornduff and Dulberger, 1978; Jesson and Barrett, 2002*a*). It is likely that early-acting inbreeding depression, causing the abortion of early developing embryos, contributed to this result, possibly in combination with partial self-incompatibility. The relative importance of these two factors affecting seed set following self-pollination are often difficult to distinguish (see Seavey and Bawa, 1986; Gibbs, 2014), and further work would be needed in *W. paniculata* to evaluate their potential contributions. However, we note that our pollination study was conducted on form 1 of *W. paniculata*, which possesses the greatest herkogamy (Fig. 3) and populations that commonly have 1:1 morph ratios (Jesson and Barrett, 2002*a*). Therefore, it seems probable that, unlike *W. multiflora* and *W. brachyandra*, this form of *W. paniculata* is largely outcrossing (and see Jesson and Barrett, 2002*a*) and therefore more likely to experience inbreeding depression than the other two highly self-compatible species.

### Weakening of negative frequency-dependent selection and breakdown of polymorphism

Floral polymorphisms, such as heterostyly and enantiostyly, are maintained in populations by negative frequency-dependent selection resulting from disassortative pollination and mating. Our study suggests that relaxation of disassortative mating resulting from increased self-pollination and assortative mating has a disruptive effect on the maintenance of equal morph ratios in *Wachendorfia* populations. We found that in populations with reduced herkogamy and, presumably, lower levels of disassortative mating, morph ratios deviated strongly from equality, consistent with a weakening of negative frequency-dependent selection. In contrast, populations with well-developed herkogamy and higher disassortative mating (and for *W. paniculata*, see Minnaar and Anderson, 2021) tended to have morph ratios much closer to the 1:1 equilibrium, as predicted with strong negative frequency-dependent selection. Although genetic estimates of disassortative mating based on parentage analysis (e.g. see Yuan *et al.*, 2023) have yet to be undertaken in *Wachendorfia*, earlier estimates of outcrossing rate using allozyme markers support the hypothesis that the degree of outcrossing in *W. paniculata* is generally higher than in *W. brachyandra* and, particularly, *W. multiflora* (Jesson and Barrett, 2002*a*), the two species with weaker herkogamy and more biased morph ratios.

Our analyses indicated that flower size, anther spacing and population size were also correlated with morph ratios, but the strength of these associations was weaker than the association with herkogamy. This finding suggests that multiple traits might co-vary during transitions to self-pollination, but that not all contribute equally to changes in mating system, a pattern also suggested by comparative analyses of heterostylous *Primula* (de Vos *et al.*, 2014). We detected no clear relationship between pollinator visitation rates and morph ratios, contrary to the expectation that insufficient pollinator service resulting in pollinator-limited seed set should destabilize the polymorphism. However, our analysis was based on relatively limited observation periods (30 h across 16 populations). In particular, most pollinator observations occurred during a single flowering season, and their duration differed between species and populations. As a result, our observations might not accurately reflect the long-term abundance of pollinators at any given site. Morph ratios represent the visible signature of cumulative mating events over many previous seasons, and given the perennial, potentially moderately long-lived life history of *Wachendorfia*, fluctuating pollinator service might result in mismatches between observed morph ratios and contemporary pollinator observations. Future studies combining long-term pollinator data with marker-based estimates of selfing and disassortative mating would help to determine whether reduced herkogamy evolves primarily as a reproductive assurance mechanism in response to chronically low pollinator visitation rates. Shifts to selfing in *Wachendorfia* could also be influenced by weak inbreeding depression and the inherent genetic transmission advantage of selfing genes, because selfers pass on two copies of their genome rather than one, as in outcrossers (Fisher, 1941; Holsinger, 1988).

Our use of quantum dots in the Langebaan population of *W. multiflora* provided insight into pollen movement within and between style morphs. Most outcross pollen transfer occurred between morphs, consistent with functional enantiostyly. However, a substantial fraction of cross-pollination was within morphs, similar to the findings of an earlier study of *W. paniculata* (Minnaar and Anderson, 2021). In contrast to the Langebaan population of *W. multiflora*, two other populations of this species (Koeberg and Redelingshuys) exhibited only self-pollen on their stigmas, demonstrating that autonomous self-pollination can replace pollinator-mediated pollen transfer when pollinator service is compromised (Barrett, 2002; Goodwillie *et al.*, 2005; Brys *et al.*, 2013). Differences between the Koeberg and Redelingshuys populations further highlight the functional role of herkogamy: Koeberg (with similar herkogamy to Langebaan, but no pollinators) received pollen loads similar to the self-pollen component deposited at Langebaan, whereas the Redelingshuys population had lower herkogamy and correspondingly higher self-pollen deposition, resulting in similar total pollen loads to Langebaan. These results confirm that reduced herkogamy increases self-pollen deposition and provide evidence for a direct mechanistic pathway linking floral architecture to potential mating outcomes.

The discovery of homostylous variants in five populations of *Wachendorfia* provides evidence that enantiostyly is evolutionarily labile and susceptible to floral modification. Homostylous individuals were restricted to populations with low herkogamy, suggesting that the breakdown of enantiostyly might be limited to the lower end of the herkogamy spectrum. Our finding of a non-linear relationship between herkogamy and self-pollination (Fig. 6) suggests that the selective advantage of homostyles occurs only once anther–stigma separation is sufficiently reduced to permit efficient self-fertilization. This parallels data from numerous heterostylous species, in which homostylous variants frequently arise in populations experiencing demographic change and pollinator-limited seed set (Charlesworth and Charlesworth, 1979; Barrett and Husband, 1990; Yuan *et al.*, 2023). In heterostylous species, the breakdown of polymorphism does not lead to a uniform outcome with respect to mating system: populations can become predominantly selfing, practice mixed mating or remain largely outcrossing, depending on a range of ecological and reproductive factors (Belaoussoff and Shore, 1995; Barrett, 2019; Yuan *et al.*, 2023). Our results suggest that enantiostylous systems follow similarly flexible pathways, with reductions in herkogamy opening multiple evolutionary routes rather than enforcing a uniform outcome involving small-flowered, strictly autogamous populations. For example, contrary to expectations under a simple selfing-transition model, some populations in our study with highly biased morph ratios still received high pollinator visitation (Supplementary Data Table S1 and S2), indicating that morph-ratio bias does not necessarily require autonomous self-pollination to maintain fertility.

Our study provides evidence that enantiostyly can undergo evolutionary breakdown through mechanisms similar to those documented in heterostylous species. By demonstrating parallel floral pathways of morph-ratio destabilization, including the emergence of homostyly and the diversification of likely mating outcomes, our findings support a general framework in which the spatial separation of reproductive organs is the key axis of phenotypic variation linking pollination ecology and the stability of floral polymorphism. In contrast to heterostyly, in which the breakdown of polymorphism has given rise to fully homostylous populations and also new species (Barrett and Shore, 1987; de Vos *et al.*, 2014; Zhong *et al.*, 2019), dissolution of enantiostyly in *Wachendorfia* appears to be at an early stage of this process, with uncertain evolutionary outcomes.

## Supplementary Material

mcag213_Supplementary_Data

## Data Availability

Raw data are provided in the Supplementary Information.

## SUPPLEMENTARY DATA

Figure S1: floral measurements taken in each *Wachendorfia* sp. population. Table S1: list of all sampled *Wachendorfia* populations with averages for herkogamy, flower width, distance between the lower anthers, distance between the side anthers, distance between the stigma and the upper anther (all in millimetres), daily display size, estimated population size, sample size for the trait measurements, morph bias, sample size for morph frequencies and GPS coordinates. Populations with homostylous variants are in bold. Asterisks indicate a proportion that is significantly different from 1:1. Table S2: list of *Wachendorfia* populations where focal pollinator observations were carried out, year of study, time spent observing (in hours), visitation rate (in visits per flower per hour), *n* the average number of flowers observed, and species visiting the flowers. Populations with homostylous variants are in bold. Table S3: pairwise comparisons of seed set between pollination treatments in three *Wachendorfia* species based on GLMs, with plant ID as random factor and Poisson error structure followed by Tukey’s *post hoc* test. Significant results are in bold.
