# Supplementary material for: Breakdown of dimorphic enantiostyly is linked to reduced herkogamy and increased self-pollination
Source: Ann Bot. 2026 Jul 17;138(4):1007–20. doi: 10.1093/aob/mcag213 (PMC13595285; doi:10.1093/aob/mcag213)
Supplement: Supplementary file 1 [file mcag213_supplementary_data.pdf]

Supplementary Information: Breakdown of dimorphic enantiostyly is linked to reduced herkogamy and increased self-pollination

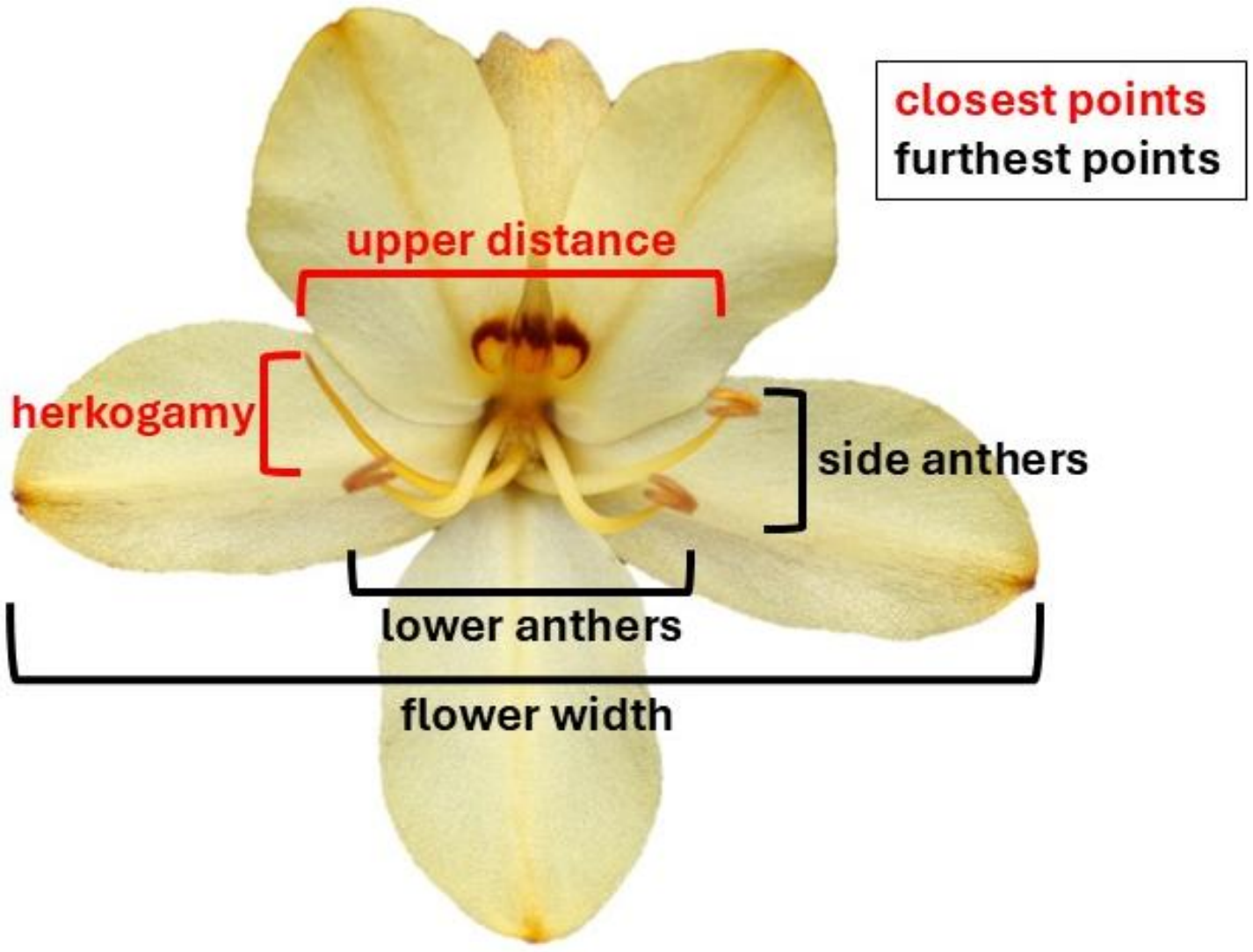

Supplementary Figure 1: Floral measurements taken in each *Wachendorfia* sp. population.

Supplementary Table 1: List of all sampled *Wachendorfia* populations with averages for herkogamy, flower width, distance between the lower anthers, distance between the side anthers, distance between the stigma and the upper anther (all in mm), daily display size, estimated population size, sample size for the trait measurements, morph bias, sample size for morph frequencies and GPS coordinates. Populations with homostylous variants in bold. Asterisks indicate a proportion that is significantly different from 1:1. Strikethrough indicates that the data was not collected.

species	population	herko-gamy	width	lower	side	upper	display size	population size	*n* floral traits	morph bias	morph ratio L:R	GPS
W. brachyandra	Capri	1.1	24.4	-	1.2	4.7	3.1	1200	20	0.06	272:344*	-34.134, 18.388
W. brachyandra	Kenilworth Racecourse	2.6	36.3	5.0	4.0	7.4	7.2	750	25	0.05	126:105	-33.998, 18.484
W. brachyandra	Lower Tokai	2.3	30.7	-	-	8.0	2.4	125	31	0.03	54:48	-34.052, 18.435
W. brachyandra	Noordhoek Common	1.7	28.8	-	-	0.7	-	275	30	0.02	96:106	-34.099, 18.373
W. brachyandra	Noordhoek Wetlands	1.4	26.6	-	2.0	5.2	4.3	175	20	0.43	113:8*	-34.108, 18.375
W. brachyandra	Pagasvlei	2.5	32.5	-	-	-	-	1000	20	0.01	132:128	-34.031, 18.429
W. brachyandra	**Rondebosch Common**	**0.6**	**24.7**	**-**	**1.5**	**2.7**	**-**	**50**	**15**	**0.18**	**60:129***	**-33.951, 18.484**
W. brachyandra	Schusterskraal	1.0	26.7	-	2.0	4.9	-	175	21	0.31	81:19*	-34.202, 18.375
W. brachyandra	Silvermine	1.0	29.1	-	-	5.0	-	40	20	0.50	0:29*	-34.088, 18.419
W. brachyandra	Silvermine Dam	0.9	28.1	-	-	-	-	50	20	0.50	50:0*	-34.078, 18.398
W. multiflora	**Banghoek**	**2.3**	**27.9**	**6.8**	**1.9**	**-**	**-**	**50**	**19**	**0.14**	**8:14**	**-32.753, 18.590**
W. multiflora	Bettys Bay	1.9	35.0	-	3.0	-	-	120	28	0.29	84:23*	-34.363, 18.884
W. multiflora	Capri	2.3	26.8	8.3	3.0	10.0	-	120	21	0.50	0:88*	-34.134, 18.388
W. multiflora	**Donkie's Kraal**	**1.5**	**23.5**	**6.3**	**4.8**	**4.1**	**-**	**35**	**10**	**0.43**	**1:14***	**-32.303, 18.536**
W. multiflora	Hopefield	2.3	22.6	6.9	1.5	-	2.7	75	20	0.10	8:12	-33.022, 18.295
W. multiflora	Imhoff farm	1.9	24.8	-	-	-	-	100	29	0.50	29:0*	-34.138, 18.350
W. multiflora	Koeberg	3.0	29.9	7.6	2.9	12.3	4.7	300	21	0.12	67:110*	-33.670, 18.456
W. multiflora	Langebaan	3.3	35.6	8.2	2.1	11.8	2.8	500	20	0.05	110:90	-33.104, 18.046
W. multiflora	Posberg	2.5	37.8	13.2	3.7	-	-	300	20	0.03	47:54	-33.103, 17.999
W. multiflora	Red Hill	1.2	/	-	-	-	3.2	-	42	0.50	42:0*	(Jesson & Barrett 2002)
W. multiflora	**Redelingshuys**	**0.7**	**24.8**	**9.1**	**2.2**	**-**	**-**	**30**	**13**	**0.39**	**2:16***	**-32.484, 18.537**
W. multiflora	Rondebosch Common	1.7	21.8	4.2	1.0	-	-	50	22	0.50	22:0*	-33.952, 18.484
W. multiflora	Salmanslaagte	3.9	37.3	13.8	3.1	-	2.7	125	39	0.01	20:19	-32.067, 19.112
W. multiflora	Shooting range	2.0	20.4	6.0	1.3	-	2.8	40	30	0.47	3:108*	-33.620, 18.474
W. multiflora	Ysterfontein	2.1	26.5	8.2	1.6	-	2.6	125	20	0.23	47:17*	-32.166, 18.788

W. paniculata	Bettys Bay	3.9	45.8	-	5.5	-	-	120	20	0.07	82:62	-34.363, 18.877
W. paniculata	Camps Bay	4.7	38.4	12.3	5.7	-	-	200	40	0.00	20:20	-33.960, 18.388
W. paniculata	Glencairn Form 1	5.5	47.9	6.4	4.1	-	5.2	500	40	0.02	429:399	-34.149, 18.426
W. paniculata	Glencairn Form 3	1.4	27.5	4.8	4.3	-	2.9	90	27	0.35	25:146*	-34.149, 18.428
W. paniculata	**Pearly Beach**	**0.8**	**28.9**	**9.5**	**3.9**	**-**	**3.0**	**1000**	**37**	**0.50**	**0:25***	**-34.678, 19.574**
W. paniculata	Jonkersdam Form 3	1.6	27.9	5.1	4.3	-	3.1	160	20	0.21	115:48*	-34.145, 18.391
W. paniculata	Kenilworth Racecourse	5.7	43.4	16.1	5.6	17.7	6.9	500	20	0.01	99:101	-33.998, 18.484
W. paniculata	Kleinmond Golf Course	4.3	37.6	-	2.4	-	-	1000	20	0.04	60:70	-34.328, 19.034
W. paniculata	Marloth Nature Reserve	6.9	42.4	17.1	5.3	22.0	-	800	20	0.01	239:245	-34.001, 20.450
W. paniculata	Retirement Home Form 1	5.7	47.6	5.4	10.5	-	5.1	440	10	0.02	213:228	-34.109, 18.401
W. paniculata	Retirement Home Form 3	1.7	36.5	2.9	5.5	-	1.8	50	30	0.48	49:1*	-34.109, 18.401
W. paniculata	Scarborough Form 3	0.9	28.3	6.1	2.2	-	2.8	20	18	0.02	11:10	-34.187, 18.376
W. paniculata	Silvermine Form 1	5.3	45.0	14.1	5.5	-	2.2	100	20	0.05	49:60	-34.111, 18.435
W. paniculata	Silvermine Form 3	2.0	27.6	10.9	4.9	-	1.8	200	19	0.39	185:23*	-34.112, 18. 429
W. paniculata	Stellenbosch Mountain	6.6	41.5	-	4.5	-	-	2000	18	0.05	136:110	-33.941, 18.887
W. paniculata	Tienieversveld	4.4	42.7	-	3.3	-	-	200	20	0.00	83:84	-33.334, 18.272
W. paniculata	Waylands	6.0	52.3	16.3	6.5	-	-	1000	40	0.00	150:149	-33.406, 18.416

Supplementary Table 2: List of *Wachendorfia* populations where focal pollinator observations were carried out, year of study, time spent observing in hours, visitation rate in visits/flower/hour, *n* the average number of flowers observed, and species visiting the flowers. Populations with homostylous variants in bold. Strikethrough indicates that no flower visitors were observed.

species	population	year	time	visitation rate	*n*	visitor species
W. brachyandra	Noordhoek Wetlands	2022	0.5	1.44	131	*Apis mellifera*, syrphid fly
W. brachyandra	Capri	2022	1.0	0.65	815	*Apis mellifera*
W. brachyandra	Rondebosch Common	2022	1.0	0.12	17	asilid fly
W. paniculata	Tienieversfeld	2022	0.8	0	20	-
W. paniculata	Stellenbosch Mountain	2022	1.6	0.47	182	*Apis mellifera, Xylocopa afra, Amegilla sp.*
W. paniculata	Waylands	2024	3.2	1.54	48	*Philoliche angulata, Apis mellifera, Australoechus hypoleucus,* hopliinid beetle
W. paniculata	Glencairn Form 1	2022	0.5	5.55	94	*Apis mellifera*, small bee
W. paniculata	Glencairn Form 3	2022	0.5	6.08	49	*Apis mellifera*, hopliinid beetle, syrphid fly
W. paniculata	Kleinmond Golf Course	2022	2.0	0.03	64	*Amegilla sp.*
W. paniculata	**Pearly Beach**	**2024**	**1.0**	**2.66**	**29**	***Apis mellifera*, small black bee**
W. multiflora	Postberg	2024	1.5	0.97	59	*Rediviva peringueyi, Rediviva parva, Anthophora wartmanni*
W. multiflora	Langebaan	2022	1.5	1.55	23	*Apis mellifera, Rediviva parva*
W. multiflora	Langebaan	2023	3.0	3.12	12	*Apis mellifera, Rediviva parva*
W. multiflora	Capri	2022	0.3	7.70	128	*Apis mellifera*
W. multiflora	Rondebosch Common	2022	0.5	0.03	57	asilid fly
W. multiflora	**Redelingshuys**	**2024**	**2.1**	**0**	**9**	**-**
W. multiflora	**Redelingshuys**	**2025**	**5.0**	**0**	**34**	**-**
W. multiflora	Koeberg	2025	4.0	0	38	-

Supplementary Table 3: Pairwise comparisons of seed set between pollination treatments in three *Wachendorfia* species based on GLMs with plant ID as random factor and poisson error structure followed by a Tukey post-hoc test. Significant results in bold.

	W. paniculata				*W.multiflora*				*W.brachyandra*			
contrast	Estimate	se	z-ratio	p	Estimate	se	z-ratio	p	Estimate	se	z-ratio	p
Intramorph cross - bagged	**1.35**	**0.26**	**5.17**	**<0.001**	0.48	0.16	2.90	0.434	0.36	0.18	1.98	0.356
Intramorph cross - intermorph cross	0.00	0.17	-0.02	1.000	-0.06	0.14	-0.40	0.999	-0.17	0.16	-1.07	0.893
Intramorph cross - de-anthered open	0.17	0.18	0.94	0.935	**0.64**	**0.15**	**4.36**	**<0.001**	0.52	0.19	2.71	0.073
Intramorph cross - hand-self	**0.58**	**0.20**	**2.95**	**0.037**	0.13	0.15	0.90	0.947	0.28	0.18	1.57	0.616
Intramorph cross - open pollination	0.03	0.16	0.16	1.000	0.06	0.12	0.46	0.998	-0.29	0.14	-2.01	0.334
Bagged - intermorph cross	**-1.35**	**0.26**	**-5.19**	**<0.001**	**-0.53**	**0.16**	**-3.32**	**0.012**	**-0.53**	**0.17**	**-3.04**	**0.029**
Bagged - de-anthered open	**-1.18**	**0.26**	**-4.50**	**0.000**	0.16	0.17	0.97	0.928	0.16	0.21	0.79	0.969
Bagged - hand-self	-0.77	0.28	-2.78	0.060	-0.34	0.17	-2.04	0.320	-0.08	0.19	-0.40	0.999
Bagged - open pollination	**-1.32**	**0.25**	**-5.20**	**<0.001**	**-0.42**	**0.15**	**-2.90**	**0.044**	**-0.64**	**0.16**	**-4.04**	**0.001**
Intermorph cross - de-anthered open	0.17	0.18	0.97	0.926	**0.69**	**0.14**	**4.89**	**<0.001**	**0.69**	**0.19**	**3.73**	**0.003**
Intermorph cross - hand-self	**0.58**	**0.20**	**2.99**	**0.034**	0.19	0.15	1.31	0.780	0.45	0.17	2.63	0.090
Intermorph cross - open pollination	0.03	0.16	0.19	1.000	0.11	0.12	0.95	0.934	-0.12	0.13	-0.90	0.948
De-anthered open - hand-self	0.42	0.20	2.08	0.298	**-0.50**	**0.15**	**-3.35**	**0.010**	-0.24	0.20	-1.18	0.845
De-anthered open - open pollination	-0.14	0.17	-0.85	0.957	**-0.58**	**0.12**	**-4.71**	**<0.001**	**-0.81**	**0.17**	**-4.70**	**<0.001**
Hand-self - open pollination	**-0.55**	**0.19**	**-2.97**	**0.036**	-0.08	0.13	-0.61	0.990	**-0.57**	**0.16**	**-3.63**	**0.004**
